# Risk factors for oral mucositis in patients with solid tumors under treatment with cetuximab: a retrospective cross-sectional study

**DOI:** 10.4317/medoral.26237

**Published:** 2023-11-22

**Authors:** Joyce Ohana de Lima Martins, Marcela Maria Fontes Borges, Cássia Emmanuela Nóbrega Malta, Janaina Motta Guerra, André Alves Crispim, Bruna Carolina Coelho, Lúcio Flávio Gonzaga Silva, Paulo Goberlânio de Barros Silva

**Affiliations:** 1 Department of Dental Clinic, Division of Oral Pathology, Faculty of Pharmacy, Dentistry and Nursing, Federal University of Ceará, Fortaleza, Ceará, Brazil; 2 Department of Dentistry, Unichristus, Fortaleza, Ceará, Brazil; 3 Hospital Haroldo Juaçaba, Ceará Cancer Institute, Fortaleza, Ceará, Brazil

## Abstract

**Background:**

This study retrospectively analyzed the risk factors for oral mucositis (OM) during cetuximab treatment.

**Material and Methods:**

We screened patients using cetuximab and retrospectively evaluated the presence of OM based on medical records. We collected information from 2 years of evaluations. Patient medical records were reviewed to obtain data on chemotherapy cycle and dose, sex, age, primary tumor, TNM stage, and head and neck radiotherapy (HNR) history. The X2 test and multinomial logistic regression were used for statistical analysis (SPSS 20.0, *p* < 0.05).

**Results:**

Among 1831 patients, OM was showed in 750 in any grade (41%), during cetuximab treatment. Most patients were female (*n*=944, 51.6%), <70years-old (*n*=1149, 62.8%), had larynx cancer (*n*=789, 43.1%) in T4 (*n*=579, 47.7%), N0 (*n*=509, 52.6%) stages. Primary tumor surgery was performed in 1476 (80.6%) patients, radiotherapy in 606 (33.1%) patients and cetuximab protocols most used involved up to four cycles (*n*=1072, 58.5%) of <400mg (*n*=996, 54.4%) cetuximab doses. Female (OR [odds ratio] = 2.17, CI95% = 1.26-3.75), >70 years-old patients (OR = 16.02, CI95% = 11.99-21.41), with HHNR (OR = 1.84, 1.41-2.40), treated with >4 cycles (OR = 1.52, CI95% = 1.16-2.01) and high doses of cetuximab (OR = 3.80, CI95% = 2.52-5.71) are the greatest risk factors for OM.

**Conclusions:**

Since the clinical benefit of cetuximab in the treatment of older patients is limited and there is a high OM, especially in women with head and neck treated with radiotherapy, high doses and a high number of cetuximab cycles must be administered with caution.

** Key words:**Mucositis, cetuximab, antibodies, neoplasm, antineoplastic agents.

## Introduction

The therapeutic protocols usually applied for cancer treatment involve surgical intervention, radiotherapy, and/or chemotherapy, but the advance in the use of neoadjuvant, adjuvant, and palliative chemotherapy has significantly increased life expectancy in the treatment of most solid tumors (1). The adjuvant treatment with monoclonal antibodies (immunotherapy) has significantly innovated traditional cancer treatment. These drugs specifically inhibit tumor growth signaling pathways, induce immune responses against tumor cells, and preserve more healthy cells, concerning traditional antineoplastic treatment protocols (2).

Human genome studies have enabled advances in technologies that detect genomic alterations at the transcription and epigenetic levels. Combining these technologies with new drugs under development constitutes the implementation of targeted therapies (3). Cetuximab is a recent drug used in monotherapy or combination with local radiotherapy. It is a monoclonal antibody directed against the epidermal growth factor receptor (EGFR) (4).

Cetuximab acts by competitively inhibiting the binding of EGFR to its ligand with consequent blockade of phosphorylation of receptor-related enzymes to suppress cell growth, induce cell apoptosis, and reduce the production of cytokines that stimulate degradation of the medium (5). Cetuximab induces apoptosis of tumor cells and keeps them in the G1 phase when they are relatively sensitive to radiotherapy. It also reduces radiotherapy-resistant S phase cells, thus increasing their sensitivity to radiotherapy treatment (6).

Still, despite its high specificity, cetuximab has been associated with dermatitis, especially oral mucositis (7). Oral mucositis occurs due to the cytotoxic effect of antineoplastic drugs on the oral mucosa. Its consequence is an erosive inflammation leading to dysphagia, taste alterations, weight loss, and secondary infections (8,9). The incidence of mucositis associated with antineoplastic chemotherapies, and their severity, depends on the antineoplastic agent, doses, and treatment time. Therefore, studies evaluating risk factors for this condition have been widely developed (10).

However, some authors have speculated that oral mucositis related to monoclonal antibodies has distinct mechanisms, apparently linked to hypersensitivity (8,11) or inflammatory vasculitis (12). In this context, knowing the risk factors for oral mucositis related to cetuximab may contribute to the understanding of its mechanisms, as well as help to understand risk groups and define prevention/treatment protocols. Thus, this study aims to evaluate risk factors for oral mucositis related to using cetuximab in patients undergoing systemic treatment for solid tumors.

## Material and Methods

- Study design and scenario

This study is a retrospective, quantitative observational cohort study guided by the STROBE initiative, an international guideline for conducting observational studies (13). This study was conducted using data on oral cavity adverse effects in patients during chemotherapy to treat solid tumors available in the electronic patient record system of the Haroldo Juaçaba Hospital/Ceará Cancer Institute (HHJ/ICC) from January 1, 2010, to December 31, 2019.

The Ethics Committee of the Haroldo Juaçaba Hospital approved this research as part of a project that includes the analysis of risk factors for adverse effects of cancer treatment in the oral cavity, whose protocol number is 4.062.135. All study phases were carried out under law 466/12 of the research ethics legislation, ensuring the confidentiality of information from the patients' medical records and keeping them until the end of the study.

- Inclusion and exclusion criteria

The inclusion criterion was patients who had performed at least one zoledronic acid infusion in the period from January 1, 201 8to December 31, 2019, at the HHJ/ICC. All drug infusions in chemotherapy services of the Unified Health System or private health insurance plans are recorded in the Tasy system with the pharmaceutical record of the active ingredient of the drug. Thus, we retrieved the services when these patients took their doses, utilizing this system.

The exclusion criteria were patients undergoing treatment for myeloproliferative disorders, occult or metastatic disease with an unknown primary site, and those with medical records lacking clinical information required to assess risk factors. Repeated patients (>1 evaluation) were also excluded.

- Socio-demographic and clinical data collection

With the number of services provided by the Tasy system's toxicity scale tool, we performed a manual search of each service's records to retrieve the clinical and pathological data of interest. Patients appearing more than once were ordered by their date of care to identify the number of chemotherapy cycles.

During the manual collection of information based on the number of care visits, the patients' medical records were collected, as well as age, sex, weight on the day of care, height, chemotherapy purpose (neoadjuvant, adjuvant, or palliative), clinical stage, chemotherapy protocol, and primary tumor location. Additionally, the tumor-node-metastasis (TNM)-2016 grading system (14) was used to classify the stage of the solid tumors. Information on previous/concomitant head and neck radiotherapy was obtained from head and neck tumors patients. All data were recorded using a Microsoft Excel spreadsheet.

- Adverse effects analysis tool

The toxicity scale tool used was the Common Terminology Criteria for Adverse Events (CTCAE). Formerly called the Common Toxicity Criteria (CTC or NCI [National Cancer Institute]-CTC), this tool contains a set of criteria for the standardized classification of adverse effects of drugs used in cancer therapy. The CTCAE system is a product of the US National Cancer Institute (NCI). It has been widely used in many clinical trials, extending beyond oncology and encoding their observations based on the CTCAE system (15).

The CTCAE system toxicity scale includes the following adverse effects: mucositis, vomiting index, diarrhea, nausea, constipation, anorexia, dysgeusia, alopecia, hand and foot syndrome, fatigue, insomnia, and dysuria. All patients were classified according to toxicity scores suggested by the Common Terminology Criteria for Adverse Events (CTCAE) v5.0 scale for adverse effects. It uses a range of grades from 1 to 5. Still, specific conditions such as dysgeusia are graded on a five-point scale: 0, absence of mucositis, grade 1 (Asymptomatic or mild), 2 (Presence of moderate pain and ulceration, without interference with food intake), 3 (severe pain with interference with food intake) or 4 (Life-threatening with the need for urgent intervention) (15).

After each medical consultation performed immediately before chemotherapy, the multi-professional team assigned the following toxicity grades for dysgeusia registered in the toxicity scale tool and exported to a standard Microsoft Excel spreadsheet containing the number and date of attendance and the degree of severity of the adverse effect. Patients with >1 evaluation were excluded.

- Statistical approach

The data were exported to IBM SPSS Statistics for Windows, Version 20.0, to perform the statistical analyses and obtain 95% confidence intervals.

The prevalence of oral mucositis grades was expressed as an absolute frequency and percentage compared to the risk factors using Fisher's exact or Pearson's chi-square tests. We included *p*<0.200 variables in a multinomial logistic regression model (multivariate analysis).

## Results

**Figure 1:**
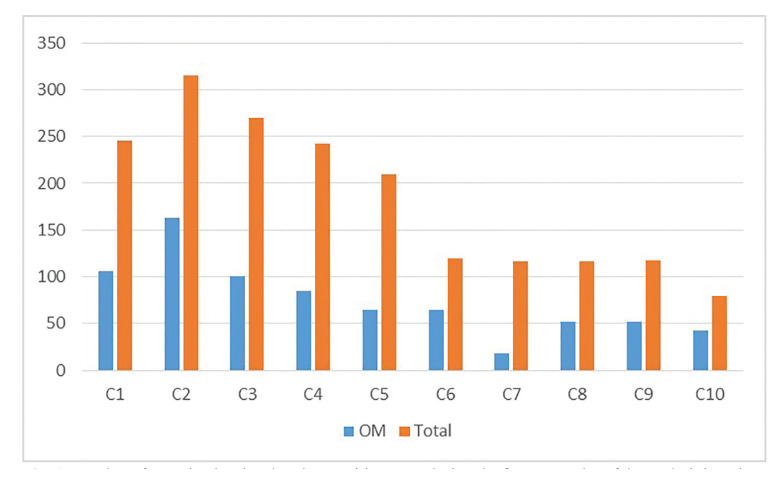
Number of cetuximab-related oral mucositis events during the first ten cycles of drug administration.

A total of 1831 patients were evaluated in this study. The prevalence of oral mucositis was 41.0%, with 750 patients experiencing this adverse effect after treatment with cetuximab (Fig. 1).

Most of the patients evaluated in female (*n*=944, 51.6%), and the mean age was 66.3±8.52 years, ranging from 42 to 81. Patients were categorized based on median age, showing 1149 (62.8%) patients aged up to 70 years and 682 (37.2%) aged over 70 years. Female gender (*p*<0.001) and age over 70 years (*p*<0.001) showed an increased prevalence of oral mucositis associated with cetuximab use (Table 1).

The most frequently treated cetuximab tumors were laryngeal (*n*=789, 43.1%) followed by colorectal (*n*=30.3%). Tumors of the mouth, glottis, and larynx had a higher prevalence of oral mucositis associated with cetuximab use (*p*<0.001) (Table 1).

Regarding staging, T4 stage (*n*=579, 47.7%) N0 stage (*n*=509,52.6%) were the most prevalent stages, and T1 (*p*<0.001) and N0/N2 (*p*<0.001) tumors showed a higher prevalence of oral mucositis associated with the use of cetuximab. Tumor resection surgery was described in most cases (*n*=1476, 80.6%) and head and neck radiotherapy in 606 (33.1%) patients, the latter being directly associated with oral mucositis (*p*<0.001) (Table 2).

**Table 1:**
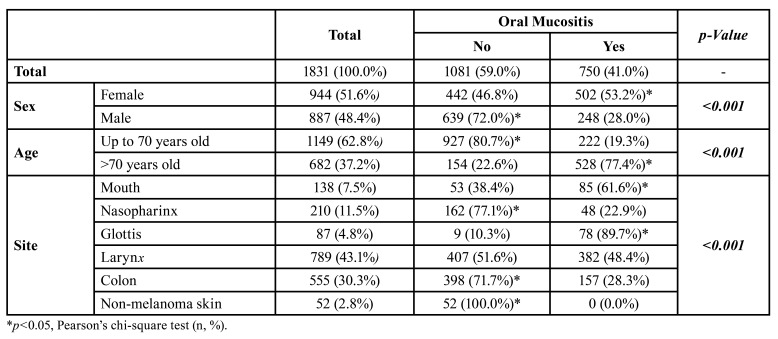
Influence of gender, age, and tumor location on the prevalence of oral mucositis in patients taking cetuximab.

**Table 2:**
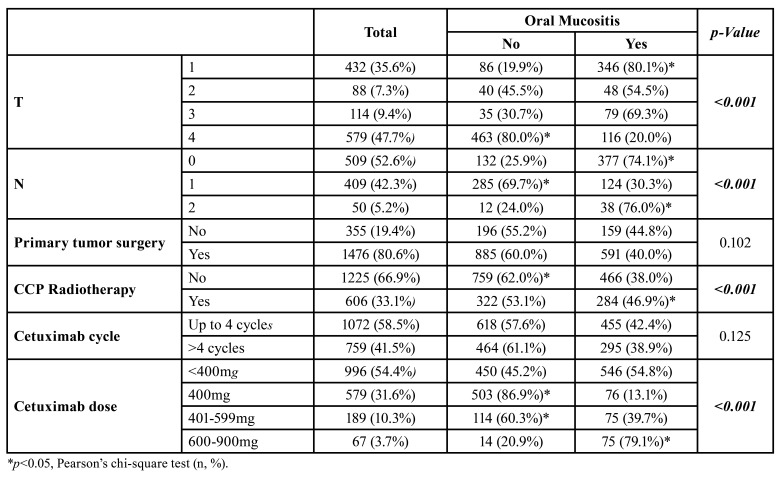
Influence of clinical features and therapeutic protocol on the prevalence of oral mucositis in patients taking cetuximab.

Most patients had two cycles of treatment with cetuximab (*n*=560, 30.6%) (Fig. 1), but the number of cycles of chemotherapy did not significantly influence the incidence of mucositis. The most frequently used doses of cetuximab were less than 400mg (*n*=996, 54.4%), and doses greater than 600mg of cetuximab significantly increased the incidence of oral mucositis (Table 2).

In multivariate analysis, age >70 years presented the highest prevalence of oral mucositis, increasing 16.02 times the chances of this outcome. The female sex showed a 2.17-fold increase in the majority of oral mucositis and head and neck radiotherapy, which increased the frequency of this outcome by 1.84 times. Cetuximab treatment cycle (<3 cycles) and dose (<400mg) showed a 1.53- and 3.80-fold increase in the frequency of oral mucositis (Table 3).

**Table 3:**
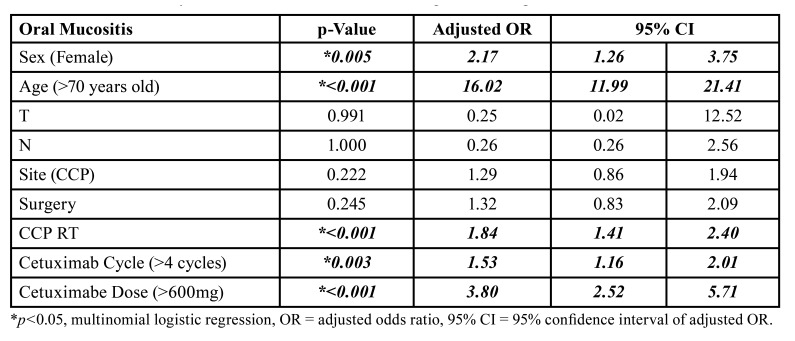
Multivariate analysis of risk factors for oral mucositis in patients taking cetuximab.

## Discussion

Treatment with cetuximab has been used as a line of therapy for colorectal tumors (16), refractory non-melanoma skin cancers (17), and in relapsed head and neck cancers (18), including as a second or first line of treatment (19). However, oral mucositis has been frankly described as a high-incidence adverse effect during the use of this monoclonal antibody (20). In our study, the frequency of oral mucositis was 41.0%, quite similar to that described in the literature. Still, this incidence depends directly on the type of tumor treated and the antineoplastic protocol used.

A recent systematic review describes that the incidence of oral mucositis is lower when monoclonal antibodies are used compared to conventional chemotherapies (21). Still, the incidence increases significantly when chemotherapy is used concomitantly (22). When used as monotherapy for colorectal cancer treatment, the incidence of oral mucositis does not exceed 10% of patients (23). During treatment for skin cancer, this adverse effect is not even mentioned as significant (24). In head and neck tumors, even when used in combination with cisplatin and/or paclitaxel, the incidence of oral mucositis related to cetuximab is 3-4.4% (23). Still, when associated with radiotherapy, the incidence increases alarmingly to values around that observed in our study (44.7%) (23-24).

We also observed a low incidence of oral mucositis in non-head and neck tumors. Similar to what is described in the literature, no cases of skin cancer and only 28.3% of colorectal tumors had oral mucositis related to treatment with cetuximab. On the other hand, patients with head and neck tumors ranged from 22.9 to 89.7% incidence of oral mucositis. The factor most strongly associated with the development of oral mucositis in these tumors is the combination of cetuximab and radiation therapy. In phase I studies, >70% of patients developed mucositis during the combination therapy (25), and these values can be as high as close to 100% (26). In one clinical trial, the incidence of oral dermatitis and mucositis combining cetuximab and radiotherapy was more elevated than conventional treatment combining cisplatin and radiotherapy (20,22,23).

Bonner (25) state that the addition of cetuximab to radiotherapy significantly improves overall survival, progression-free survival, and loco-regional control if compared to radiotherapy alone. However, treatment with cetuximab seems to show more benefit in tumors with high EGFR expression (>40%) (26). Hence, although treatment with cetuximab combined with radiotherapy compared to radiotherapy alone does not significantly interfere with the quality of life of patients being treated for head and neck tumors (27), its use should be considered based on the overall health status of the patient since the addition of cetuximab to conventional treatments significantly increases the incidence of non-hematologic adverse effects, mainly cutaneous/dermal such as skin rash, hand and foot syndrome, and oral mucositis (28,18).

Other factors that emerged as risk factors for the appearance of OM, with relevance in this study, were gender and age. The highest prevalence of mucositis was found in females and patients over 70 years of age. Although Nishii *et al*. (2020) evaluating patients with head and neck cancers showed a higher incidence of oral mucositis in men, several studies have shown that women have an increased risk of oral mucositis during chemotherapy (29). Female hormones show a complex and sometimes controversial association with local and systemic inflammatory processes and somehow contribute to this increase (30).

As for age, individuals over 70 years of age have a higher severity of oral mucositis, but there is no cause related to this. In general, young and elderly patients show the same clinical benefit during systemic treatment, but the fragility of health in older patients significantly increases the risk of toxicity (25). In the present study, age was the major risk factor for oral mucositis related to treatment with cetuximab, and attention should be paid to this fact, as it may be necessary to consider the use of cetuximab in older patients given the increased adverse effects (27,28,30).

Severe oral mucositis leads to treatment discontinuation due to painful symptomatology of the lesions and compromises treatment prognosis (22,23). In the multivariate analysis of our study, we showed that sex, amount, and size of the dose but especially age are important risk factors for oral mucositis. Alongi (19) and Bonner (25) describe that the increase in survival of patients with head and neck cancers treated with RT and cetuximab is only modest for patients over 70 years old, so possibly the use of cetuximab in these specific groups (women, over 70 years old and requiring high doses and a large number of cycles) should be highly considered.

Perhaps the most significant limitation of our study is its retrospective nature. It makes it impossible to track important information about prognosis and other adverse effects. However, this study shows critical risk factors to patients taking cetuximab, which may help clinical management during oncological treatment, especially head and neck tumors.

## References

[1] Vanneman M, Dranoff G (2012). Combining immunotherapy and targeted therapies in cancer treatment. Nat Rev Cancer.

[2] Kimiz-gebologlu I, Gulce-iz S, Biray-avci C (2018). Monoclonal antibodies in cancer immunotherapy. Mol Biol Rep.

[3] Zhang Y, He D, Zhang W, Xing Y, Guo Y, Wang F (2020). ACE inhibitor benefit to kidney and cardiovascular outcomes for patients with non-dialysis chronic kidney disease stages 3-5: a network meta-analysis of randomised clinical trials. Drugs.

[4] Gillison ML, Trotti AM, Harris J, Eisbruch A, Harari PM, Adelstein DJ (2019). Radiotherapy plus cetuximab or cisplatin in human papillomavirus-positive oropharyngeal cancer (NRG Oncology RTOG 1016): a randomised, multicentre, non-inferiority trial. Lancet.

[5] Vincenzi B, Zoccoli A, Pantano F, Venditti O, Galluzzoet S (2010). Cetuximab: from bench to bedside. Curr Cancer Drug Targets.

[6] Mazzarella L, Guida A, Curigliano G (2018). Cetuximab for treating non-small cell lung cancer. Expert Opin Biol Ther.

[7] Moon C, Chae YK, lee J (2010). Targeting epidermal growth factor receptor in head and neck cancer: lessons learned from cetuximab. Exp Biol Med.

[8] Daugėlaitė G, Užkuraitytė K, Jagelavičienė E, Filipauskas A (2019). Prevention and treatment of chemotherapy and radiotherapy induced oral mucositis. Medicina.

[9] Lalla RV, Saunders DP, Peterson DE (2014). Chemotherapy or radiation-induced oral mucositis. Dent Clin North Am.

[10] Pulito C, Cristaudo A, La Porta A, Zapperi S, Blandino G, Morrone A (2020). Oral mucositis: the hidden side of cancer therapy. J Exp Clin Cancer Res.

[11] Elyasi S, Hosseini S, Moghadam MRN, Aledavood SA, Karimi G (2016). Effect of oral silymarin administration on prevention of radiotherapy induced mucositis: A randomized, double‐blinded, placebo‐controlled clinical trial. Phytother Res.

[12] Holt MH, Liu V, Fairley J (2018). Medium-vessel vasculitis presenting as multiple leg ulcers after treatment with abatacept. JAAD Case Rep.

[13] Malta M, Cardoso LO, Bastos FI, Magnanini MMF, Silva CMFP (2010). STROBE initiative: guidelines on reporting observational studies. Rev Saúde Pub.

[14] Treanor CJ, McMenamin UC, O'Neill RF, Cardwell CR, Clarke MJ, Cantwell M (2016). Non-pharmacological interventions for cognitive impairment due to systemic cancer treatment. Cochrane Database Syst Ver.

[15] Ikesue H, Yamamoto H, Hirabatake M, Hashida T, Chung H, Inokuma T (2022). Risk Factors of Proteinuria in Patients with Hepatocellular Carcinoma Receiving Lenvatinib. Biol Pharm Bull.

[16] Meads C, Round J, Tubeuf S, Moore D, Pennant M, Baylisset S (2010). Cetuximab for the first-line treatment of metastatic colorectal cancer. Health Technol Assess.

[17] Kalapurakal SJ, Malone J, Robbins KT, Buescher L, Godwin J, Rao K (2012). Cetuximab in refractory skin cancer treatment. J Cancer.

[18] Mei M, Chen YH, Meng T, Qu LH, Zhang ZY, Zhang X (2020). Comparative efficacy and safety of radiotherapy/cetuximab versus radiotherapy/chemotherapy for locally advanced head and neck squamous cell carcinoma patients: a systematic review of published, primarily non-randomized, data. Ther Adv Med Oncol.

[19] Alongi F, Bignardi M, Garassino I, Pentimalli S, Cavina R, Mancosu P (2012). Prospective phase II trial of cetuximab plus VMAT-SIB in locally advanced head and neck squamous cell carcinoma. Strahlenthe Onkol.

[20] Peña-cardelles JF, Salgado-Peralvo AO, Garrido-Martínez P, Cebrián-Carretero JL, Pozo-Kreilinger JJ, Moro-Rodríguez JE (2021). Oral mucositis. Is it present in the immunotherapy of the immune checkpoint pd1/pd-l1 against oral cancer? A systematic review. Med Oral Patol Oral Cir Bucal.

[21] Soutome S, Yanamoto S, Nishii M, Kojima Y, Hasegawa T, Funahara M (2021). Risk factors for severe radiation-induced oral mucositis in patients with oral cancer. J Dent Sci.

[22] Dote S, Itakura S, Kamei K, Hira D, Noda S, Kobayashi Y (2018). Oral mucositis associated with anti-EGFR therapy in colorectal cancer: single institutional retrospective cohort study. BMC Cancer.

[23] Maubec E, Petrow P, Scheer-Senyarich I, Duvillard P, Lacroix L, Gelly J (2011). Phase II study of cetuximab as first-line single-drug therapy in patients with unresectable squamous cell carcinoma of the skin. J Clin Oncol.

[24] Preneau S, Rio E, Brocard A, Peuvrel L, Nguyen JM, Quéreux G (2014). Efficacy of cetuximab in the treatment of squamous cell carcinoma. J Dermatolog Treat.

[25] Bonner JA, Harari PM, Giralt J, Azarnia N, Shin DM, Cohen RB (2006). Radiotherapy plus cetuximab for squamous-cell carcinoma of the head and neck. N Engl J Med.

[26] Walsh L, Gillham C, Dunne M, Fraser I, Hollywood D, Armstrong J (2011). Toxicity of cetuximab versus cisplatin concurrent with radiotherapy in locally advanced head and neck squamous cell cancer (LAHNSCC). Radiother Oncol.

[27] Vermorken JB, Mesia R, Rivera F, Remenar E, Kawecki A, Rottey S (2008). Platinum-based chemotherapy plus cetuximab in head and neck cancer. N Engl J Med.

[28] Curran D, Giralt J, Harari PM, Ang KK, Cohen RB, Kies MS (2007). Quality of life in head and neck cancer patients after treatment with high-dose radiotherapy alone or in combination with cetuximab. J Clin Oncol.

[29] Li J, Yan H (2018). Skin toxicity with anti-EGFR monoclonal antibody in cancer patients: a meta-analysis of 65 randomized controlled trials. Cancer Chemother Pharmacol.

[30] Vokurka S, Bystrická E, Koza V, Scudlová J, Pavlicová V, Valentová D (2006). Higher incidence of chemotherapy induced oral mucositis in females: a supplement of multivariate analysis to a randomized multicentre study. Support Care Cancer.

